# Experimental evidence of the neurotoxic effect of monosodium glutamate in adult female Sprague Dawley rats: The potential protective role of *Zingiber officinale* Rosc. rhizomes

**DOI:** 10.1016/j.sjbs.2023.103824

**Published:** 2023-10-05

**Authors:** Samah A. El-Hashash, Mohamed A. El-Sakhawy, Hanan S.E. Eldamaty, Abdullah A. Alqasem

**Affiliations:** a Department of Nutrition and Food Science, Faculty of Home Economics, Al-Azhar University, Nawag, Tanta City, P.O. Box 31732, Egypt; b Department of Medical Laboratory Sciences, College of Applied Medical Sciences, Prince Sattam bin Abdulaziz University, Al-Kharj 11942, Saudi Arabia; c Department of Medicinal and Aromatic Plants, Desert Research Center, Cairo, Egypt

**Keywords:** *Zingiber officinal*e rhizomes, Neurotoxicity, Oxidative stress, Inflammation, Lipid profile, Monosodium glutamate, Rats

## Abstract

Strategies to prevent the health abnormalities associated with the extensive use of MSG (monosodium glutamate) as a flavoring booster are badly needed. The current study was conducted to investigate oxidative stress, inflammation, and abnormal lipid profile as the main risk factors of neurotoxicity in MSG-exposed female albino rats. Besides, the effect of concurrent consumption of *Zingiber officinale* rhizomes powder was studied at low doses. Twenty rats (total) were split into 4 separate groups. The 1st group was a negative control group (without any treatment), while the others received 6 mg MSG/kg. The 2nd group was left untreated, whereas the 3rd and 4th groups were given a regular laboratory diet that included ginger rhizome powder supplements (GRP, 0.5 & 1%, respectively) for six weeks. In brain tissue homogenates, exposure to MSG caused a significant depletion of gamma-aminobutyric acid (GABA) and total protein levels, while triglycerides and cholesterol contents were significantly elevated. Moreover, a noteworthy upsurge in oxidative load and inflammation markers was also noticed associated with a marked reduction of antioxidant levels, which histopathological staining verified further. The rat diet formulated with GRP, with a dose-dependent effect, resulted in increased GABA and total protein contents and attenuated inflammation, oxidative stress, abnormal lipid profile, and marked histological changes in cerebral cortical neurons of MSG-administered animals. Therefore, this study reveals that GRP shields rats against the neurotoxicity that MSG causes. The anti-inflammatory as well as antioxidant, and lipid-normalizing properties of rhizomes of ginger may be accountable for their observed neuroprotective action.

## 1 Introduction

One of the main contributors to cell death and malfunction in many illnesses is oxidative stress (OS). Chronic stress may induce cognitive decline, disruption of synaptic plasticity, and neurodegeneration, according to the etiology of many neurodegenerative disorders. Oxidative stress is particularly harmful to the cortical and hippocampal regions of the brain (Saleem et al., 2020). In the mammalian central nervous system (CNS) as well as the human CNS, the primary excitatory neurotransmitter is glutamate (Glu). Numerous typical brain functions, including memory, cognition, and learning, are supported by Glu. Additionally, Glu plays a fundamental part in controlling bio-energetic processes, for instance, the citric acid cycle, gluconeogenesis, glycolysis, and ketone body formation. Additionally, it serves as a precursor for glutathione (GSH) and gamma-aminobutyric acid (GABA). Moreover, it is one of the primary determinants of both excitotoxic neuronal cell damage and central pain transduction processes (Hudspith, 1997, Prinsen et al., 2017).

However, excessive brain Glu levels–induced neurotoxicity was highly reported, which is known as excitotoxicity. Glu toxicity was found to induce neuronal dysfunction and oxidative stress in many neurodegenerative pathologies, for instance, Huntington’s, Alzheimer’s, and Parkinson’s diseases, additionally to seizure disorders (Kostandy, 2012, Yap et al., 2020). However, based on the type of cell and the availability of glutamate receptors, Glu induces toxicity to varying degrees (Kritis et al., 2015). It is noteworthy that the brain tissue involving the hippocampus has many Glu receptors (Dief et al., 2014). Although Glu is not only found naturally in some foods but also synthesized in the body (Kurihara, 2009, Reitzer, 2009), excess amounts of it in the body often come from high consumption of monosodium glutamate (MSG)–containing food products. MSG (E621), the sodium salt of glutamic acid, has been added to foods that are highly consumed worldwide in recent years (Niaz et al., 2018). MSG is utilized to intensify the natural flavors of a wide range of food products by the name “China salt”, and it might be present in packaged foods even if it isn't listed on the label (Alalwani, 2014). It provides an exclusive taste to prepared foods which are also known as a “savoury” taste and in Japanese is known as “umami” (Xiong et al, 2009). The European standard recorded 10 g/kg (one percent) of product for MSG. Other products may include this additive within permitted limits, but it's possible to consume a lot of these items on a daily basis without being aware of the overall quantity of MSG consumed. Unfortunately, the consumption of MSG-containing products continuously increasing in some developing countries particularly those geared for children such as noodles, flavorings for potato chips, et cetera. Therefore, the European standard decided the safety of glutamic acid and its derivatives intake limit of 30.0 mg/kg B.W/day. In a modern study, MSG consumed in European countries ranged from 8 to 198 mg/kg B.W/day (Banerjee et al., 2021, Ayed et al., 2023, Yu et al., 2023).

Despite being permitted by the FDA, it was shown that a high MSG intake was linked to several diseases, including obesity caused by cancer, asthma, and diabetes. Additionally, MSG consumption was linked to genotoxicity, renal toxicity, hepatotoxicity, and reproductive toxicity (Kazmi et al., 2017). Neurotoxic effects of MSG were highly studied and evidenced in both young (Quines et al., 2014, Hazzaa et al., 2020) and adult rats (Atef et al., 2021, Albrakati, 2023).

Free radicals are responsible for the various secondary complications in all diseases including neurotoxicity. This fact generated the approach of the prevention/treatment of diseases using antioxidants (Saleem et al., 2020). So, synthetic antioxidants were used widely. Given the most recent concerns that synthetic antioxidants have a negative impact on human health, a bad need for screening safer and more effective antioxidants for these diseases from natural sources was found. In recent years, more attention is paid to plants including fruits, cereals, nuts, and vegetables, in addition to medicinal herbs for use as neuroprotective therapeutics. They are the richest source of antioxidants like phenolic compounds, flavonoids, gallotannins, and other related polyphenols.

Ginger (*Zingiber officinale* L.) has been consumed for numerous centuries as food and alternative medicine. It is a native of Southeast Asia and a member of the plant family Zingiberaceae (Liu et al., 2019). Nowadays, ginger is known to have anti-inflammatory, antioxidant, and anti-apoptotic characteristics. Several earlier research has also shown that it is useful in both treatment and prevention of gastrointestinal, neurological, pulmonary, and cardiovascular illnesses (Semwal et al., 2015, Sahardi and Makpol, 2019, Ozkur et al., 2022). The plant's edible portion is the rhizome. The bioactive substances found in the rhizome of ginger, for instance, gingerols, zingiberene, shogaols, and paradols, are thought to be responsible for its medicinal benefits (Liu et al., 2019, Kausar et al., 2021).

In search of such safe and available antioxidant, anti-inflammatory, and hypolipidemic agents for the prevention of MSG-induced neurotoxicity in adult female rats, ginger rhizomes in the powder form, mimicking the image used as a seasoning by most housewives, have been tested at low concentrations in the current study.

## 2 Materials and methods

### 2.1 Plant materials and preparations

Ginger as fresh rhizomes were obtained in December 2022 from a shop of herbs and medicinal plants located in Tanta City, Egypt. The plant was systematically classified as *Zingiber officinale* Rosc. by taxonomists in the Herbarium of the Botany Dep., Faculty of Science, Cairo University, Cairo, Egypt.

Fresh rhizomes were dried in the shade after being divided by a knife into small pieces to accelerate the drying, then it was ground with a hammer mill (Thomas Digital ED-5 Wiley Mill, Germany) to a fine powder (GRP), and were sieved with a screen of 2–3 mm pore size. Pulverized were kept in dry tightly closed dark glass jars until used.

### 2.2 Chemicals and biochemical kits

Pure white MSG crystals were obtained from Sigma Aldrich Co., and kits used for biochemical test investigation were purchased from Gamma Trade Co. Other additional chemicals were purchased from El-Gomhouria Company for Drugs, Cairo, Egypt.

### 2.3 Animals, housing and experimental diet

Adult 20 female albino animals of Sprague_Dawley rat strain weighed 150 ± 5 g were bought from the animal colony, Vaccine and Immunity Organization, Helwan, Cairo, Egypt. Experimental animals were used for the biological tests in line with the Laboratory Animal Care and Use Guide for scientific purposes, National Committee for Research, and accepted by the Animal Ethics Committee of Science Faculty, Tanta University (IACUC-SCI-TU-0324). All laboratory biological specimens and hazardous wastes were disposed of safely.

Animals were kept individually in well-ventilated cages under a controlled environment in a space that is kept at an appropriate humidity level, 22–25 °C, and twelve hours of light, and darkness, with free access to water and standard pelleted laboratory feed. Concentrate combination (10%), molasses (3%), yellow corn (49%), sunflower oil (15%), wheat bran (10%), soybean meal (11%), table salt (0.5%), CaHPO_4_ (0.1%), crushed limestone (0.2%), lysine (0.2%), mineral-vitamin premix (0.3%), and dl-methionine (0.7%) were all included in the diet. The animal food was bought from the Agricultural Development Co., 6th of October, Giza, Egypt.

### 2.4 Experimental design

After the acclimation period of one week, the 20 rats were split into 4 groups, each containing five rats. The 1st group was kept as a negative control group, while the other three (2nd −4th) groups received 6 mg of MSG per kilogram of body weight each day through a stomach tube. The 2nd group was left untreated as a positive control, the 3rd and 4th groups were given the regular laboratory diet along with 0.5 and 1%, respectively, of GRP for six weeks. The entire experiment involved giving MSG and GRP to rats at the same time.

The six-week experiment was conducted. The animals were given unlimited access to food and water (*ad-libitum*), and their weight was measured once a week. Each rat's MSG dosage was altered by varying its body weight. MSG dose was selected since previous studies in rat models (Ibrahim et al., 2011, Okon et al., 2020) used it.

### 2.5 Tissue processing methods

#### 2.5.1 Tissue collection and distribution

After the experimental test was completed, rats were fasted overnight and anesthetized using Anahal before being sacrificed. The whole brain from each rat was extracted through careful dissection, washed 3 times in cold (0 °C) sterilized physiological saline, blotted separately on ashless filter paper, and then dichotomized into two specimens. The first specimen, from the cerebrum specifically, was immersed in neutral formalin (10 %) solution buffered for subsequent examination of the histopathology, while the other specimen, taken from the brain as a whole, was frozen with liquid nitrogen and kept at −80 °C until used.

#### 2.5.2 Preparation of tissue homogenates

The other specimen was weighed and grinded with a Potter-Elvehjem tissue grinder (20–30 strokes, up and down), then divided into 4 parts. To estimate the amounts of GABA, the first component part was well-mixed in cold saline (0 °C) and centrifuged for 10 min at 10,000 rpm at 0 °C.

The second part was used for lipids estimation. Lipids were extracted and purified as described by Betancor et al. (2019). Tissue sample (0.2 g) was blended with 1.8 mL of two mixed solvents 2:1 (v/v) of chloroform–methanol. The resulting homogenate was then put directly into Eppendorf tubes (2 mL). After standing at −20 °C for 10 min, the homogenate centrifugation for 10 min at 2400 rpm. The solution has two phases, an upper aqueous layer (25%) of methanol (polar part) and a lower layer (75%) chloroform (nonpolar part). The chloroform layer was aspirated into a clean tube, and it was then thoroughly rinsed with 0.2 mL of KCl (0.05 M) before being centrifuged for 10 min at 2400 rpm. Two layers were separated into the clean Eppendorf tubes, then were kept frozen (- 20 °C) until needed for further studies.

For protein estimation, the third part was used and homogenized in cold 0.9% saline at a ratio of 1:10 (w/v) utilizing a high-speed homogenizer. The homogenate sample samples were centrifuged for 20 min at 2000 rpm. They were then separated into 1 mL portions and kept at −20 °C until use for further tests.

For studies on oxidative stress and lipid peroxidation, exactly 0.2 g of whole brain tissue (the 4th part) were individually homogenized in homogenizing cold (0 °C) 1.8 mL buffer adjusted to 7.2 pH using a Teflon pestle homogenizer. After being placed into a 2 mL Eppendorf microtube, the entire contents were centrifuged for 10 min at 3000 rpm. Until needed for further use, the supernatant was kept at − 20 °C.

### 2.6 Biomarkers measurement

#### 2.6.1 GABA determination

GABA level was assayed in brain tissue homogenate by using the fluorophotometric method described by Wagih (2012).

#### 2.6.2 Lipids and total protein quantitation

Triglycerides (TG) and cholesterol (Cho.) were determined in brain tissue homogenates using the method of Betancor et al. (2019), with some modifications. For TG quantification, using an Ecotherm (heating/cooling dry bath), 50 Âµl of the lipid extract were transferred into an Eppendorf tube, and then evaporated until dry at 55 °C. Then, ethyl alcohol (200 μL) was added and vortexed. After that, the tubes were filled with 1 mL of the reconstituted reagent (triacylglycerol), stirred, and incubated for 5 min at 37 °C. After 60 min, the absorbance at 546 nm was measured against the reagent blank.

As for cholesterol (Cho.), using an Ecotherm (heating/cooling dry bath), 50 Âµl of the lipid extract were transferred into an Eppendorf tube and then evaporated until dry at 55 °C. Then, a mixture (20 μL) of chloroform and triton-X100 (1:1, v/v) was added and vortexed. After that, the tubes were filled with 1 mL of cholesterol reagent kit (Randox assay), stirred, and incubated for approximately 5 min at 37 °C. After 60 min, the absorbance at 546 nm was measured against the reagent blank.

For total protein (TP) determination, the ultraviolet method (UV 260/280 nm) was used as explained by Stoscheck (1990).

#### 2.6.3 Oxidative load and inflammation markers determination

The reduced glutathione levels were evaluated with the method illustrated by Siddiqui et al. (2021), while activities of the catalase (CAT), and glutathione-S-transferase (GST) were assayed using the methods of Toledo et al. (2011). On the other hand, nitric oxide (NO) and the peroxidation marker of the lipids (MDA, malondialdehyde) were quantified following the referenced methods of Miranda et al. (2001) and Fu et al. (2018), respectively.

Moreover, the tumor necrosis factor-alpha (TNF-α) was estimated by Quantikine ELISA Immunoassay kit (R&D systems) as per the guidelines provided by the manufacturer. The main antibody was used to sensitize 96-well microplates for 30 min at room temperature. The tissue sample was then added, cultured for a further 30 min, and washed. After washing, a peroxidase-conjugated secondary antibody was applied and allowed to incubate. Then, an ELISA plate reader was used to calculate the cytokine concentration.

### 2.7 Histopathological assessment

Cerebral specimens were fixed for 24 h in formalin solution at 22 ± 2 °C before being dehydrated in ethanol in increasing grades, cleaned in terpineol, and embedded in paraffin wax. Haematoxylin and Eosin (H&E) stain was used to mount and stain sections that were five millimeters thick (Bancroft and Layton, 2013). The histological changes in each preparation were microscopically inspected.

### 2.8 Statistical analysis

Statistical computer software (Version 20; Untitled - SPSS Data Editor) was applied to do the statistical analysis. An analysis of variance (ANOVA) test was used to classify the data in one way. Mean ± standard deviation (mean ± SD) was used to express the results. Duncan's test at *p* < 0.05 was used to determine whether the variations in means were significant.

## 3 Results

### 3.1 GABA content

The mean value of GABA in the whole brain tissue homogenate of the MSG-exposed group was statistically significantly (*p*-value < 0.05) lower than the control group. MSG-exposed rats which were fed diets supplemented with GRP (0.5 and 1%) reported significant elevations (*p* < 0.05). Moreover, the higher the concentration of GRP, the higher the level of GABA (Table 1).

**Table 1:** Effect of GRP at two concentrations on GABA level in whole brain tissue homogenates of MSG -exposed.

**Groups Parameters**	**Control**	**MSG -exposed**	**MSG -exposed + 0.5% GRP**	**MSG -exposed + 1% GRP**	***P* value**
**GABA (µg/g tissue)**	219.30 ± 27.44^c^	130.00 ± 16.45^a^	177.40 ± 22.33^b^	200.00 ± 24.84^bc^	0.000

The results are displayed as mean ± SD. Significance level at *p* < 0.05. The small letter “a” refers to the smallest value. Similar letters in each row imply partial or complete insignificance. MSG; Monosodium glutamate, GABA; Gamma aminobutyric acid, GRP; Ginger rhizomes powder.

### 3.2 Lipids and total protein content

The effect of GRP at two concentrations on triglycerides, cholesterol, and total protein levels in whole tissue (brain) homogenates of MSG-exposed vs. control group was explained in Table 2. It was detected that triglycerides and cholesterol contents of brain tissue homogenates of the MSG-exposed group were significantly (*p*-value < 0.05) higher than those of the control group. In contrast, total protein content showed a significant decrease. The inclusion of GRP in diets of MSG–exposed rats resulted in a reduction in the levels of both triglycerides and cholesterol, while total protein content was elevated. GRP showed a dose-dependent effect. Accordingly, its improving effects were more significant in MSG–exposed group receiving the high concentration (1%).

**Table 2:** Effect of GRP at two concentrations on triglycerides, cholesterol, and total protein levels in whole brain tissue homogenates of MSG-exposed.

**Groups Parameters**	**Control**	**MSG -exposed**	**MSG -exposed + 0.5% GRP**	**MSG -exposed + 1% GRP**	***P* value**
**TG (mg/g tissue)**	175.97 ± 16.82^a^	271.87 ± 28.74^c^	241.87 ± 24.95^bc^	213.87 ± 21.71^b^	0.000
**Cho. (mg/g tissue)**	229.07 ± 23.72^a^	292.53 ± 32.96^b^	262.33 ± 26.28 ^ab^	231.32 ± 23.72 ^a^	0.005
**TP (mg/g tissue)**	40.39 ± 3.83^c^	28.44 ± 2.86 ^a^	35.22 ± 2.93^b^	35.68 ± 3.38^b^	0.000

The results are displayed as mean ± SD. *p* < 0.05 is considered significant. The small letter “a” refers to the smallest value. Similar letters in each row imply partial or complete nonsignificance. GRP; Ginger rhizomes powder, MSG; Monosodium glutamate, TG; Triglycerides, Cho.; Cholesterol, TP; Total protein.

### 3.3 Oxidative load and inflammation markers

In Table 3, a significant reduction (*p*-value < 0.05) in the level of the antioxidant compound GSH was observed in whole brain tissue homogenates of the MSG-exposed group compared to that of the control group. Similarly, the activities of the antioxidant enzymes, GST and CAT, were also lowered significantly. Conversely, the levels of NO and MDA as oxidative markers as well as TNF-α as an inflammatory marker showed significant increments. Feeding MSG–exposed rats on GRP–supplemented diets improved their antioxidant and anti-inflammatory status. As a result, the levels of NO, MDA, and TNF-α were significantly reduced. Herein, GRP also showed a dose-dependent effect.

**Table 3:** Effect of GRP at two concentrations on oxidative load and inflammation markers in whole brain tissue homogenates of MSG -exposed.

**Groups Parameters**	**Control**	**MSG -exposed**	**MSG -exposed + 0.5% GRP**	**MSG -exposed + 1% GRP**	***P* value**
**CAT (U/g tissue)**	1.50 ± 0.19^c^	0.57 ± 0.07 ^a^	0.76 ± 0.10^b^	0.84 ± 0.11^b^	0.000
**GSH (mmol/g tissue)**	1.03 ± 0.13 ^d^	0.35 ± 0.04 ^a^	0.70 ± 0.08^b^	0.83 ± 0.10^c^	0.000
**GST (U/g tissue)**	5.70 ± 0.71 ^d^	1.99 ± 0.25 ^a^	3.93 ± 0.48^b^	4.73 ± 0.58^c^	0.000
**MDA (nmol/g tissue)**	55.23 ± 6.91 ^a^	137.27 ± 17.16^d^	98.00 ± 12.15^c^	73.33 ± 9.37^b^	0.000
**NO (µmol/g tissue)**	37.10 ± 4.61 ^a^	78.50 ± 9.74^c^	58.53 ± 7.57^b^	52.13 ± 6.88^b^	0.000
**TNF-α (pg/mg protein)**	50.12 ± 5.39 ^a^	170.30 ± 17.20^d^	125.00 ± 12.96^c^	90.05 ± 9.29^b^	0.000

The results are displayed as mean ± SD. *p* < 0*.*05 is considered significant. The small letter “a” refers to the smallest value. Similar letters in each row are implying partial or complete nonsignificance. GRP; Ginger rhizomes powder, MSG; Monosodium glutamate, CAT; Catalase, GST; Glutathione-S-transferase, GSH; Reduced glutathione, NO; Nitric oxide, MDA; Malondialdehyde, TNF-α; Tumor necrosis factor alpha.

### 3.4 Histopathological examination

**Fig. 1:**
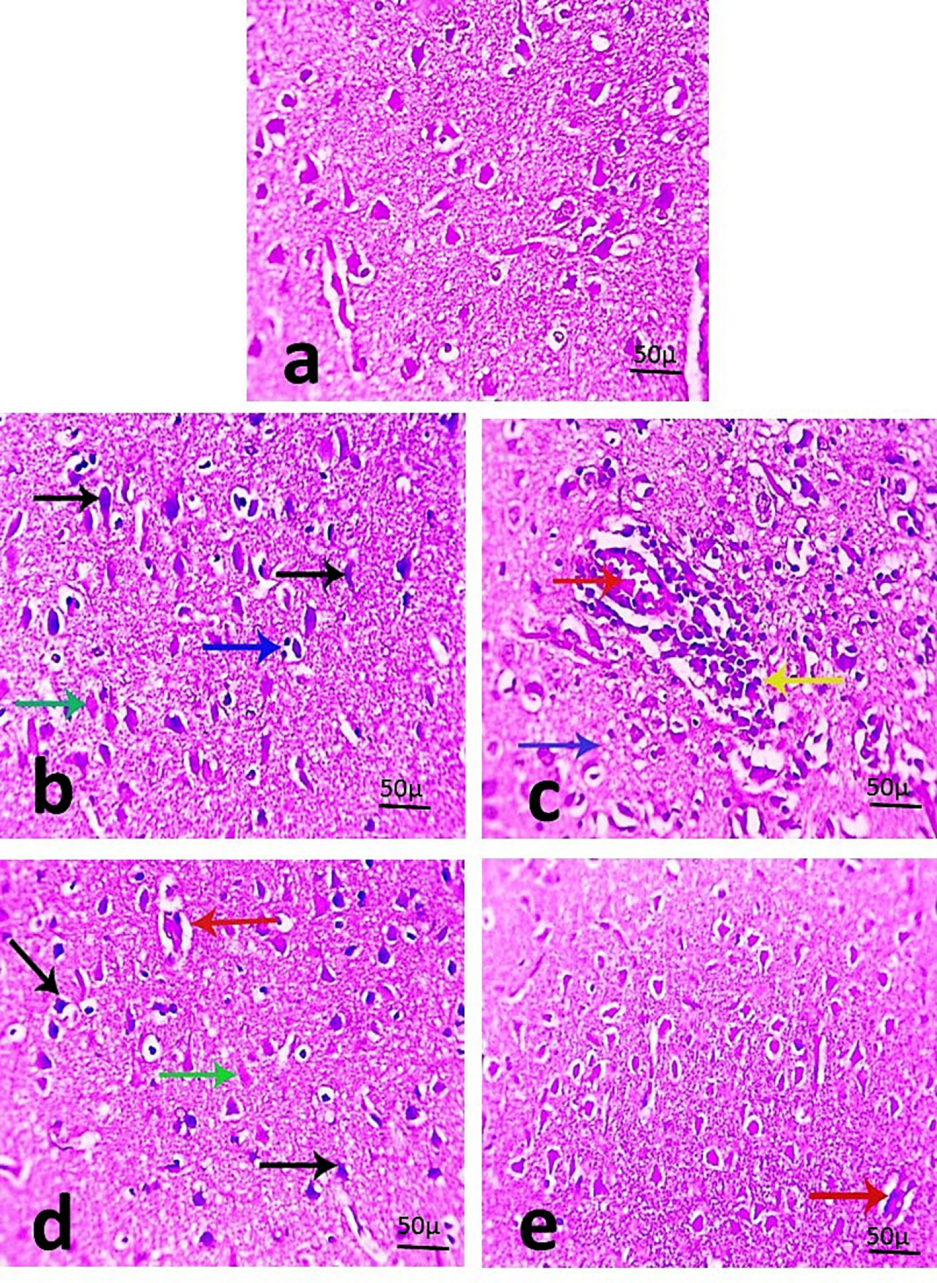
**(a-e).** Microscopic pictures of stained cerebral sections explaining normal cortical neurons in the control group (Figure a). Cerebral sections from MSG–the exposed group showing marked changes in the cortex including severe degeneration in neurons labeled by black arrows (characterized by shrinkage and more eosinophilic cytoplasm), some necrotic neurons lose their nuclei and have pale eosinophilic cytoplasm (green arrow), satellitosis (blue arrows), markedly congested blood vessels (red arrow) with prominent perivascular lymphocytic aggregation labeled by yellow arrow (Figures b & c). Cerebral sections from the MSG + 0.5% GRP-received group showing degenerated (black arrows) and necrotic (green arrow) neurons with mildly congested blood vessels labeled by the red arrow (Figure d). Cerebral sections from MSG + 1% GRP–received group showing improved histological picture of neurons with mildly congested blood vessels labeled by red arrow (400 × bar 50) (Figure e).

The examination of hematoxylin and eosin-stained cerebral sections from the standard control group (1st group) exhibited typical normal cortical neurons (Fig. 1a). MSG with no herbal intervention induced marked cortex abnormalities including severe degeneration in neurons (shrinkage and increased eosinophilic cytoplasm), satellitosis, and markedly congested blood vessels with prominent perivascular lymphocytic aggregation. Moreover, some necrotic neurons lost their nuclei and have pale eosinophilic cytoplasm (Fig. 1b & c). Cerebral sections from the MSG + 0.5% GRP–received group showed some improvement, as only some degenerated and necrotic neurons were noticed with mildly congested blood vessels (Fig. 1d). More improvement was noticed in cerebral sections from the MSG + 1% GRP–received group, where a recovered histological picture of neurons with mildly congested blood vessels was observed (Fig. 1e).

## 4 Discussion

Food additives are very important in the food industry. However, studies revealed the harmful effects associated with overexposure to a great number of them. So, periodical evaluation is necessary. The high oxygen consumption and lipid-rich content of the brain elevate its susceptibility to oxidative stress, leading to impaired normal central nervous system functions (Salim, 2017). MSG was proven to stimulate oxidative and inflammatory reactions and impair brain functions (Shivasharan et al., 2013).

According to the present results, a significant decrease of brain GABA levels in MSG–exposed rats was noticed. This result agreed with the result of Campos- Sepúlveda et al. (2009) who claimed that MSG neurotoxicity lowers glutamic acid decarboxylase activity in some parts of the central nervous system and, hence, GABA content, evidencing increased possibility of suffering from neurological and psychiatric illnesses. MSG exposure, in the current study, induced a fat accumulative effect and decreased protein content of rat brain which was previously supported by recent studies. Of them, Uti et al. (2020) reported that phospholipids, total lipids, cholesterol concentrations, and triacylglycerol in brain tissue of MSG-obese Wistar rats were considerably (p-value *<* 0.05) greater than the control group's usual levels. Moreover, Depciuch et al. (2022) evaluated the biochemical changes induced in the brain tissue of MSG-exposed rats and found that compared to the control group, the albumin level of the MSG group was significantly lower (*p* < 0.05). It was supposed that MSG can give rise to oxidative stress and directly and indirectly protein modification (Mustafa et al., 2017).

Oxidative damage due to biochemical interactions between target molecules and reactive oxygen species (ROS), for instance, proteins, lipids, and nucleic acids, is mainly correlated to the etiology and progression of various neurodegenerative ailments. Similarly, inflammation has the same effect. The oxidative damage and inflammation augmented by MSG in the brain tissue were previously proved in rats (Shivasharan et al., 2013, Uti et al., 2020, Kumar et al., 2021, Depciuch et al., 2022). Herein, the concentrations of MDA, the lipid peroxidation biomarker, nitric oxide (NO), and TNF –α, as an inflammation marker, were significantly elevated (p *<* 0.05) in brain tissue homogenates of MSG -administered rats in comparison to the standard control group. In this regard, Uti et al. (2020) reported strong positive correlations in brain tissue in both thiobarbituric acid reactive substances (TBARS) compared to total lipids and TBARS compared to total cholesterol, while Kumar et al. (2021) stated that MSG caused a noteworthy upsurge of TNF-α levels in the brain of rats (*p* < 0.001). As for NO level, it was explained that mitochondrial malfunction ultimately improves cytokine production, which causes stimulation of genes; for instance, inducible nitric oxide synthase (iNOS), which consequently raises generation of NO, associated with higher NO in current findings and resulting in additional damage of mitochondria (Farombi and Onyema, 2006). Concomitant to these findings, a significant reduction in brain GSH levels as well as GST and CAT antioxidant enzyme activities was observed in the MSG-administered group in current findings, and these results agreed with those had previously been reported by (Shivasharan et al., 2013, Onaolapo et al., 2016, Uti et al., 2020, Depciuch et al., 2022). Reduction in GSH is a sign of tissue aging, and the degree of reduction correlates with the degree of injury (Hussein et al., 2015).

In general, the histopathological changes noticed in the brain matter (tissue) of the MSG-intoxicated group were in line with the results of many studies (Shivasharan et al., 2013, Onaolapo et al., 2016, Abd El-Wahed et al., 2019). The ability of MSG to improve ROS production resulted in oxidative injury demonstrated by decreased antioxidant levels and/or activities, and enhanced lipid peroxidation, as displayed herein, is the mechanism by which it gives rise to these anomalies in brain structure, as suggested in many studies. Abnormal lipid profile and inflammatory stimulating actions may be other causes for such histological changes.

In existing research, GRP, especially with its high concentration (1%), corrected GABA, lipids, and protein levels. Besides, it improved both antioxidant and anti-inflammatory defense systems in brain tissue homogenate. Due to these actions, structure abnormalities disappeared quietly. The results of numerous investigations using various models of neurotoxicity (Akinyemi and Adeniyi, 2018, Farag et al., 2019) rather than MSG (Hussein et al., 2017) boosted the preventive qualities of ginger rhizomes. Across these investigations, the phenolic and flavonoid compounds found in ginger rhizomes such as 6-gingerol and its derivatives, 6-shogaol, and 6-paradol, in addition to zingerone and β-bisabolene were the bioactive protective agents (Soliman et al., 2018, Sapkota et al., 2019). The most prevalent and efficient of them in dried form was 6-shogaol (Bak et al., 2012, Park et al., 2013).

In general, *Z. officinale* has many potential mechanisms of action according to the bioactive effects. For instance, *Z. officinale* has been shown to help with hyperlipidemia and hyperglycemia by modulation of transcription factors such as nuclear factor-kβ, peroxisome proliferator-activated receptors, and adenosine monophosphate-activated protein kinase mediates these positive effects. High antioxidant capability of *Z. officinale* active compounds directly scavenging free radicals and triggering the Nrf2 signaling. Importantly, the excess production of nitrogen reactive species or reactive oxygen species is thought to be a cause of neurodegenerative diseases, and antioxidants in ginger play an important role in their prevention. Furthermore, *Z. officinale* active compound mechanism as anti-inflammatory may be attributed to a decrease of proinflammatory cytokines. It can prevent inflammatory responses by decreasing NF-kβ, which results in decreased cytokine gene expression (Mao et al., 2019, Arcusa et al., 2022).

## 5 Conclusion

Ginger is anticipated that it will lessen oxidative stress indicators and inhibit histopathological alterations in the brain. These results shed light on ginger's possible neuroprotective abilities and its capacity to mitigate the harmful outcomes of MSG on the central nervous system. The current findings boost the influence of *Zingiber officinale* rhizomes powder (taken with diet) as a neuroprotective agent caused by its anti-inflammatory and antioxidant in addition to lipid normalizing efficiencies.

MSG-induced neurodegenerative disorders were reduced by ginger rhizomes powder at low doses. Therefore, more research is needed to improve our understanding on the role and mechanism of ginger rhizomes to overcome and prevent monosodium glutamate negative effects. In addition, it is recommended to isolate the ginger's active compounds and carry out further molecular analysis to understand its molecular mechanism.

## Declaration of competing interest

The authors declare that they have no known competing financial interests or personal relationships that could have appeared to influence the work reported in this paper.
